# Suicide Mortality Risk among Patients with Lung Cancer—A Systematic Review and Meta-Analysis

**DOI:** 10.3390/ijerph20054146

**Published:** 2023-02-25

**Authors:** Luisa Hofmann, Michael Heinrich, Hansjörg Baurecht, Berthold Langguth, Peter M. Kreuzer, Helge Knüttel, Michael F. Leitzmann, Corinna Seliger

**Affiliations:** 1 Department of Psychiatry and Psychotherapy, University Hospital, Ludwig-Maximilians-University, Nußbaumstraße 7, 80336 Munich, Germany; 2 Department of Psychiatry and Psychotherapy, University of Regensburg, Universitätsstraße 84, 93053 Regensburg, Germany; 3 Faculty of Medicine, University of Regensburg, Universitätsstraße 31, 93053 Regensburg, Germany; 4 Department of Epidemiology and Preventive Medicine, University of Regensburg, Franz-Josef-Strauß-Allee 11, 93053 Regensburg, Germany; 5 University Library, University of Regensburg, Universitätsstraße 31, 93053 Regensburg, Germany; 6 Department of Neurology, University Hospital Heidelberg, Im Neuenheimer Feld 400, 69120 Heidelberg, Germany

**Keywords:** suicide, cancer, lung cancer, meta-analysis, systematic review, SMR

## Abstract

The risk for suicide in patients with cancer is higher compared to the general population. However, little is known about patients with lung cancer specifically. We therefore implemented a systematic review and random-effects meta-analysis of retrospective cohort studies on suicide in patients with lung cancer. We searched a high number of common databases up to 02/2021. For the systematic review, a total of 23 studies was included. To exclude bias due to patient sample overlap, the meta-analysis was performed on 12 studies. The pooled standardized mortality ratio (SMR) for suicide was 2.95 (95% Confidence Interval (CI) = 2.42–3.60) for patients with lung cancer as compared to the general population. Subgroups with a pronouncedly higher risk for suicide compared to the general population were found for patients living in the USA (SMR = 4.17, 95% CI = 3.88–4.48), with tumors of late stage (SMR = 4.68, 95% CI = 1.28–17.14), and within one year after diagnosis (SMR = 5.00, 95% CI = 4.11–6.08). An increased risk for suicide was found in patients with lung cancer, with subgroups at particular risk. Patients at increased risk should be monitored more closely for suicidality and should receive specialized psycho-oncological and psychiatric care. Further studies should clarify the role of smoking and depressive symptoms on suicidality among lung cancer patients.

## 1. Introduction

Patients with cancer are exposed to massive psychosocial distress [1]. Correspondingly, depressive symptoms occur more frequently in patients with malignant diseases [2,3], and several studies claimed increased suicide rates in cancer patients (summarized in [4]). Lung cancer has been shown to be associated with particularly severe psychological distress [5]. Recent population-based studies indicated that lung cancer is associated with an increased risk of suicide compared to the general population [6,7,8,9,10]. Furthermore, suicide mortality risk in lung cancer patients ranked among the highest of all cancer types in several studies [11,12]. Meanwhile, cancers of the respiratory tract are considered the most frequent cause of cancer-related death worldwide [13], linked to an overall five-year survival rate of around 16%, even in highly industrialized countries such as the USA [14]. There are several different types of lung cancer with distinct etiologies, treatment options and prognoses [15]. Therefore, we believe that an in-depth analysis of suicide risks and risk factors specifically for patients with lung cancer is warranted.

By identifying individuals at highest risk for suicide, specific screening tools for suicidality can be established and implemented into the clinical routine to decrease suicide-associated mortality. This is highly relevant considering a cancer-associated suicide rate of 27.5 per 100,000 person years [16].

## 2. Materials and Methods

### 2.1. Eligibility Criteria, Systematic Literature Search, and Data Extraction

In analogy to our previous study [4], we included observational longitudinal cohort studies for our systematic review on lung cancer and suicide. Studies had to be based on cancer patients as cases and non-cancer patients as the control group. The primary outcome of studies to be included was the incidence of suicide, quantified as suicide rates, observed numbers of deaths or standardized mortality ratios (SMRs). Secondary outcomes included subgroup analyses on risk factors for suicide in lung cancer patients. Lung cancer was defined according to the International Classification of Disease (ICD-10-C34.), coded in national cancer registries. Studies had to comprise 95% confidence intervals (CI). Articles in non-English language, editorials, letters and conference abstracts were excluded.

We performed a systematic literature search according to the PRESS [17] and MOOSE [18] as well as the PRISMA guidelines [19]. The protocol of the study on overall suicide mortality among patients with cancer has been registered to PROSPERO under CRD42021265254. Briefly, the databases EMBASE (Ovid, from 1974), MEDLINE (Ovid, from 1946), PsycINFO (EBSCOhost, from 1884), Science Citation Index Expanded and Social Sciences Citation Index (Web of Science, from 1965 and1990, respectively), CINAHL (EBSCOhost, from 1981) and Google Scholar were searched from the date of inception until February 2021. In addition, we screened reference lists of the included studies for additional relevant reports. Full details of the search, including search strategies and a PRISMA-S checklist [20], are published elsewhere [4]. An update of the literature search up to December 2022 retrieved no additional studies to be considered in the meta-analysis.

Records retrieved from the database searches were collected using the reference management software Endnote (version X9) and de-duplicated according to the method described by Bramer et al. [21].

**Figure 1:**
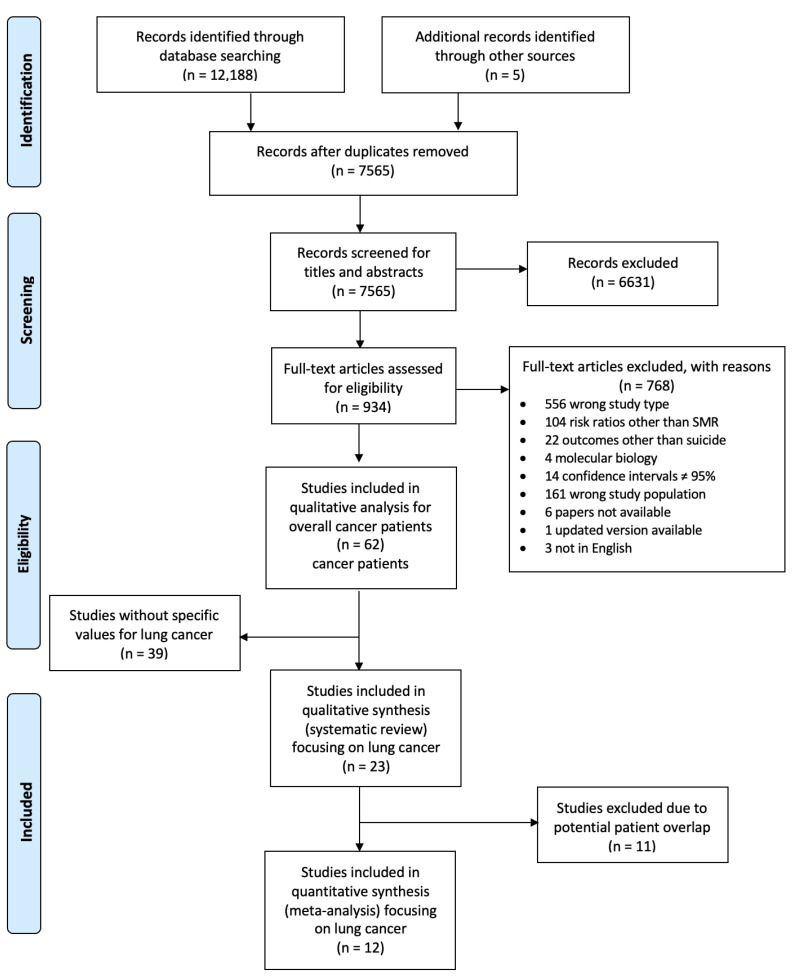
PRISMA Flow Diagram for study selection. Abbreviations: *n*, number of studies.

Two authors (M.H., L.H.) performed the study selection independently, according to the PRISMA flowchart (Figure 1) by first screening titles and abstracts, then full texts. A third researcher acted as consultant for disagreements.

Subsequently, M.H. and L.H. extracted study data, including first author, year of publication, study population size, number of suicide cases, geographic region, type of study, length of follow-up, tumor type, further subgroups, relative risk estimates, CIs and all available adjustment factors.

For missing SMRs, we calculated those values based on indicated observed and expected patient numbers.

Quality assessment followed the Newcastle–Ottawa scale (NOS) [22], classifying studies with ≥7 points as high quality studies.

For the systematic review, we used all 23 studies. However, facing the risk of potential patient overlap due to studies from the same cancer registries with overlapping recruiting time periods [23], in an additional analysis we excluded 11 studies, prioritizing studies with larger amounts of different subgroup stratifications and arrived at 12 studies for the meta-analysis.

The final set of extracted data were organized in Microsoft Excel (Version 16) worksheets.

### 2.2. Statistical Analyses

Using a random-effects model, we calculated the log(RRi) with its standard error of si = di/1.96 (di as the maximum of [log(upper 95% CI bound of RRi) − log(RRi)] and [log(RRi) − log(lower 95% CI bound of RRi)]).

Overall CIs were calculated using the Mid-P exact method by R-Studio [24]. Overall effects were estimated using random-effects models with estimated between-study variance $\tau^{2}$ by restricted maximum-likelihood estimate (REML) [25].

To investigate suicide rates among lung cancer patients according to risk factors such as sex, age, geographic region, ethnicity, marital status, histology, time after diagnosis, and stage, we carried out subgroup analyses and random effects meta-regression. For multiple test correction, we applied the false discovery rate (FDR) method, and corrected results are presented as *q*-values [26].

We calculated potential study heterogeneity using Q-statistics and I^2^ statistics and we examined possible publication bias by funnel plots, Begg’s [27] and Egger’s tests [28].

All statistical analyses were carried out using the packages ‘metafor’ in R (version 4.1.1) [29].

## 3. Results

### 3.1. Study Selection and Characteristics

We retrieved 12,188 records from the electronic databases, with five studies being added through other sources. After deduplication, we screened 7565 publications for titles and abstracts. In a second step, full texts were screened. Among 62 studies on suicide in cancer patients, we found 23 studies that presented specific data on patients with lung cancer [6,7,8,9,10,11,12,16,30,31,32,33,34,35,36,37,38,39,40,41,42,43,44]. Whereas 12 studies provided data on 1,790,027 patients with lung cancer, 11 studies gave only numbers of lung cancer patients who committed suicide, or risk estimates for suicide, but not the total number of patients with lung cancer. During follow-up, 5498 patients with lung cancer committed suicide.

Most studies (*n* = 12) used data from Europe, followed by the US (*n* = 7) and Asia (*n* = 4). Further characteristics are summarized in Table 1.

### 3.2. Main Analysis

**Figure 2:**
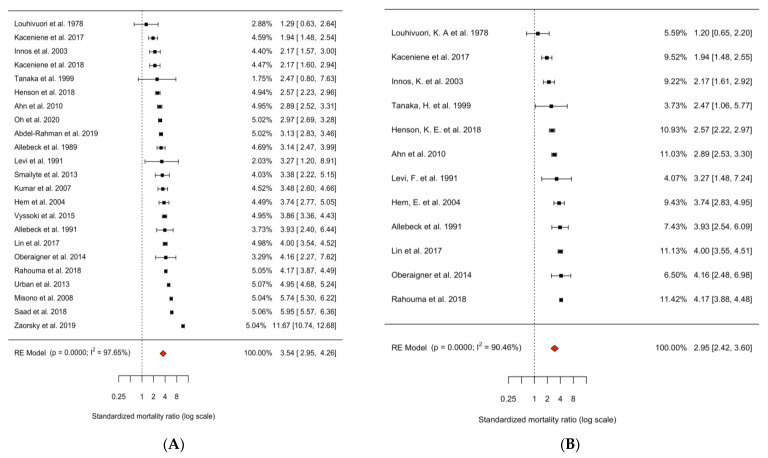
Forest plot of random effects meta-analysis of cohort studies of suicide in patients with lung cancer compared to the general population. (**A**) All 23 cohort studies (of suicide in lung cancer patients), I^2^ = 97.76%, P-heterogeneity < 0.0001. (**B**) 12 non-overlapping cohort studies (of suicide in lung cancer patients), I^2^ = 90.46%, P-heterogeneity < 0.0001. All studies were sorted by risk estimate and weighted for their contribution to the summary risk estimate. Abbreviations: SMR, standardized mortality ratio; CI, confidence interval [6,7,8,9,10,11,12,16,30,31,32,33,34,35,36,37,38,39,40,41,42,43,44].

In all 23 studies, we found a significantly elevated risk of suicide in lung cancer patients compared to the general population (SMR = 3.54, 95% CI = 2.95–4.26, *p*-value < 0.0001; Figure 2A). After exclusion of studies with potential patient overlap, the suicide mortality risk was increased 2.95-fold compared to the general population (SMR = 2.95, 95% CI = 2.42–3.60, *p*-value < 0.0001; Figure 2B). Both analyses showed considerable heterogeneity among studies (I^2^ = 97.65% and 90.46%, both *p*-values for heterogeneity < 0.0001).

**Figure 3:**
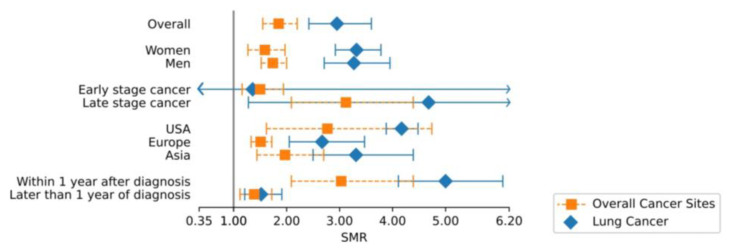
Comparison of overall and subgroup SMR of lung vs. all other cancer sites. Abbreviations: SMR, standardized mortality ratio; CI, confidence interval. Exact values of SMR and CI are indicated separately.

To identify potential causes of heterogeneity, we carried out stratified analyses. In addition to the suicide mortality risk analysis of patients with lung cancer compared to the general population, we compared the SMR of patients with lung cancer to the SMR of pooled cancer sites [4] to capture potential lung-cancer-specific differences across population subgroups. Across all subgroups, in patients with lung cancer, suicide mortality risk was increased compared to other pooled cancer sites (2.95, 95% CI = 2.42–3.60 vs. 1.85, 95% CI = 1.55–2.20) (Figure 3).

**Figure 4:**
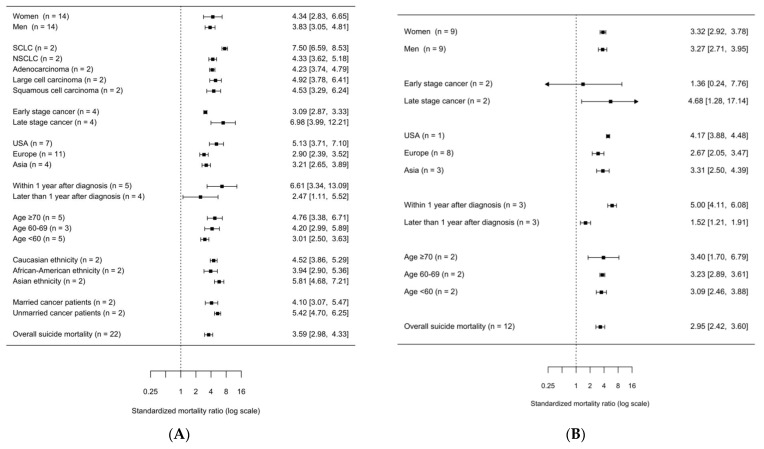
Summary forest plot of subgroup analyses of suicide in patients with lung-cancer. (**A**) All 23 studies sorted by risk estimate. I^2^ = 85.70%, P-heterogeneity = 0.0000. (**B**) 12 studies sorted by risk estimate. I^2^ = 90.29%, P-heterogeneity = 0.0000. Abbreviations: SMR, standardized mortality ratio; CI, confidence interval; n, number of studies included in the analysis.

We also explored subgroups of lung cancer patients at particular risk for suicide. Figure 4 shows a summary of specific subgroups based on data obtained from at least two independent studies. 

### 3.3. Subgroup Analyses

After exclusion of studies with potential patient overlap, the risk of suicide according to time after diagnosis was analyzed, showing a significantly higher (*p*-value < 0.001) suicide risk within one year after diagnosis (SMR = 5.00, 95% CI = 4.11–6.08) as compared to more than one year after diagnosis (SMR = 1.52, 95% CI = 1.21–1.91).

In further subgroup analyses, no statistically significant differences in suicide risk were observed (Figure 4B and Appendix B, Table 1). In all nine studies that stratified by gender, both men and women with lung cancer showed an elevated SMR for suicide compared to the general population (SMR for women = 3.32, 95% CI = 2.92–3.78; SMR for men = 3.27, 95% CI = 2.71–3.95). Age-specific differences were investigated in two studies [16,42]. Risk for suicide in patients with lung cancer was slightly increased across different ages at diagnosis (SMR for <60 years of age = 3.09, 95% CI = 2.46–3.88, SMR for 60–69 years of age = 3.23, 95% CI = 2.89–3.61, SMR for ≥70 years of age = 3.40, 95% CI = 1.70–6.79). For patients with lung cancer living in the USA, the most accentuated risk of suicide (SMR = 4.17, 95% CI = 3.88–4.48) was observed compared to those in Asia (SMR = 3.31, 95% CI = 2.50–4.39) or Europe (SMR = 2.67, 95% CI = 2.05–3.47). Patients with metastasized tumors were at elevated risk (SMR = 4.68, 95% CI = 1.28–17.14), whereas patients with a regional cancer stage showed a non-significantly lower SMR of 1.36 (95% CI = 0.24–7.76).

Further meta-regression analyses are shown in Appendix B Table A1. 

We performed additional subgroup analyses with the full set of 23 studies, considering the risk of potential patient sample overlap (Figure 4A). Two studies with potential patient overlap investigated marital status, histological subtype, and ethnicity. Married cancer patients tended to have a lower risk of suicide (SMR = 4.10, 95% CI = 3.07–5.48) compared to unmarried patients (SMR = 5.42, 95% CI = 4.70–6.25).

Patients with small-cell lung cancer were at a higher risk of committing suicide (SCLC; SMR = 7.50, 95% CI = 6.59–8.54) than patients with non-small-cell lung cancer (NSCLC; SMR = 4.33, 95% CI = 3.62–5.18), adenocarcinoma (SMR = 4.23, 95% CI = 3.74–4.78), large cell carcinoma (SMR = 4.92, 95% CI = 3.78–6.40) and squamous cell carcinoma (SMR = 4.53, 95% CI = 3.29–6.24).

Regarding ethnicity, in African American lung cancer patients, a tendency to lower risk of suicide was observed (SMR = 3.94, 95% CI = 2.90–5.35) compared to Caucasians (SMR = 4.52, 95% CI = 3.87–5.28) or other ethnicities (SMR = 5.81, 95% CI = 4.67–7.23).

### 3.4. Assessment of Publication Bias and Study Quality

**Figure A1:**
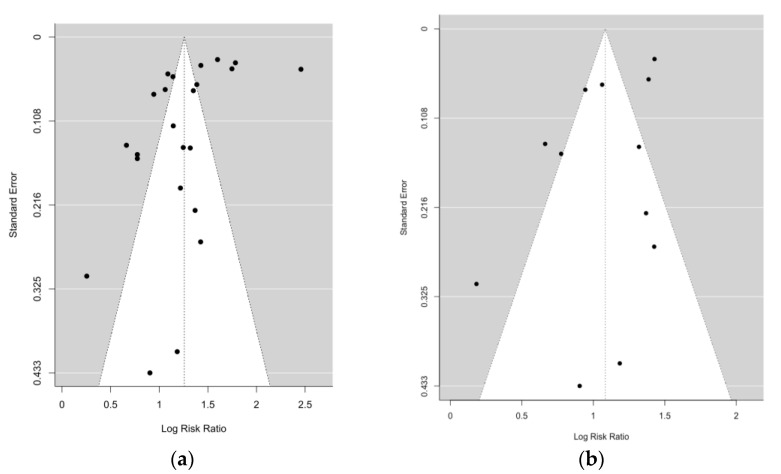
(**a**) Funnel plot for random effects meta-analysis including 23 risk estimates of suicide in lung cancer patients. *p*-value for Begg’s test = 0.7147; *p*-value for Egger’s test = 0.0214. (**b**) Funnel plot for random effects meta-analysis including 12 risk estimates of suicide in lung cancer patients. *p*-value for Begg’s test = 0.6384; *p*-value for Egger’s test = 0.3328.

The funnel plot (Appendix A Figure A1a) showed some asymmetry around the pooled SMR estimate of all 23 studies, regardless of their potential sample overlap. While formal testing using Begg’s correlation test revealed no significant deviation from symmetry (*p*-value for Begg’s test = 0.7147), Egger’s regression test suggested significant asymmetry (*p*-value for Egger’s test = 0.0214). Restricting the analyses to studies with no overlapping samples, neither Begg’s correlation (*p*-value = 0.6384) nor Egger’s regression test (*p*-value = 0.3328) nor visual inspection (Appendix A Figure A1b) indicated publication bias. All 23 studies were considered high quality according to the Newcastle–Ottawa scale (NOS) by three independent researchers [4].

## 4. Discussion

The aim of this study was to examine SMRs for suicide in patients with lung cancer and to identify subgroups with a particularly high risk. We found an overall elevated suicide rate among lung cancer patients compared to the general population, but also almost twice as high as for other cancer sites combined [4]. The increased suicide risk in lung cancer patients was also explored in subgroups defined by sex, age, geographic region, ethnicity, marital status, tumor type and stage, and time since diagnosis.

In most cases, lung cancer is associated with a fatal prognosis [45] and it is often diagnosed at a late stage [46]. Common symptoms of lung cancer are dyspnea, fatigue, pain and nausea [47]. Those symptoms may lead not only to physical but also psychological and social restrictions, which may be more severe than for other cancer sites [48,49]. It becomes increasingly difficult to follow usual habits and take part in everyday social life, as normal functions such as eating and talking and eventually breathing become impaired or painful. Therefore, the severity of the disease and its symptoms likely contribute to increased risk for suicidality [50]. However, suicide rates are also elevated among lung cancer patients with a fair prognosis [16]. 

Altogether, there is only limited knowledge about the reasons, that contribute to the increased risk for suicide among lung cancer patients [51]. One might speculate that reasons for increased suicidality in lung cancer patients include a lack of psychological support and lack of empathic communication by healthcare teams, possibly due to time restrictions and staff shortages in clinical settings [52]. Furthermore, there may be a lack of knowledge on specific risk factors for suicide [53]. In addition, patients with lung cancer often suffer from stigmatization. A qualitative research study postulated that fear of stigmatization and exclusion precluded lung cancer patients from utilizing screening options, seeking support, or informing doctors, families and friends [54]. Even if healthcare standards are high, there could be a lack of social support [55]. Lung cancer patients, especially former or current smokers, but also never-smokers, may also feel a sense of shame [54] given the fact that campaigns to quit smoking in the media have become more and more popular, while lung cancer awareness strategies show minimal effects when compared to breast cancer [56]. Moreover, smokers may feel themselves responsible for all nicotine-associated damage. Facing the fear of being judged, but also self-blaming by this unplanned ‘side-effect’ leads to a notably increased stress response [51,57]. 

The data presented in our study are derived from three different parts of the world, each of which has a different suicide prevalence rate and factors leading to suicide. By prioritizing SMRs over other types of risk estimates, we sought to minimize bias from country-specific preexisting differences in suicidality in general.

In general, tumors of the respiratory system are more common in men, who—independently from a cancer diagnosis—bear a higher risk for suicide than women [58]. In our analysis, which was based on age and gender-adjusted SMRs, we observed an equally elevated risk for committing suicide in both men and women. In addition, the lung cancer subtype SCLC, which has the worst prognosis of all subtypes, is more often seen in men than women [59].

Apart from exploring gender-associated risks, we also explored further subgroups of lung cancer patients to identify patients at particular risk.

Time after diagnosis has previously been a strong risk factor for suicide in general cancer patients according to earlier investigations [4]. Within the first year after diagnosis, suicide rates were pronouncedly high. This could be explained by the acute stress and anxiety reaction caused by being diagnosed with a potentially fatal cancer [60]. Furthermore, the change from “normal life” to “life with lung cancer” is associated with various adaptations which may appear overwhelming when coping strategies have not been sufficiently established.

In terms of geography, a particularly high SMR was observed in the USA. Patients in the USA lack universal health insurance care. The for-profit industry leads to higher health care costs, especially for cancer treatment [61,62,63], but does not necessarily result in equal outcomes [64,65,66]. Thus, it may be more difficult for US patients to receive adequate support for their physical and mental needs. In particular, lung cancer patients who often have additional severe pre-existing conditions, such as chronic obstructive pulmonary disease, could be concerned about the economic burden imposed on themselves and their families. Notably, in the first phase after diagnosis, treatment for lung cancer is associated with pronouncedly high therapeutic costs [67,68].

The strongest variation in risk (although based on analyses with potential patient overlap) was detected in subgroups defined by lung cancer stage and histology. Metastatic disease was a prominent risk factor for suicidality in lung cancer patients. Lung cancer most often spreads to the bones, kidneys and the CNS, which leads to pain and dysfunction of the musculoskeletal and neurological systems [69]. Tumor-associated pain at baseline was found to be significantly associated with suicidal intentions [70]. In addition, metastatic growth is known to lower therapeutic options, implicating a worse prognosis, which may lead to feelings of hopelessness, depression and eventually suicidality.

Patients with small-cell-lung-cancer (SCLC) had a more than seven-fold elevated risk of committing suicide compared to the general population, which was also significantly increased when compared to patients with non-small-cell-lung-cancer, adenocarcinoma, large cell carcinoma and squamous cell carcinoma. One possible explanation is the exceptionally poor prognosis, with more limitations in everyday and social life due to the frequent occurrence of paraneoplastic and organic brain syndromes (e.g., limbic encephalitis) in patients with SCLC [71], which are often associated with psychopathologic symptoms [72]. By comparison, in patients with other lung cancer subtypes with poor prognosis, such as adenocarcinoma, lower suicide rates were observed. Adenocarcinoma more often occurs in females and non-smokers [73,74,75,76]. This suggests that smoking status may play a more crucial role. SCLC is associated with the use of tobacco [77], which is linked to higher levels of depression and anxiety [78]. Previous investigations claimed associations of tobacco smoking with both depression and schizophrenic psychosis [79,80,81], and both disorders are related to an increased risk of suicide [82]. In addition, there is an increased suicidality in smokers independently from depression [83]. Since smoking is considered the number one cause of lung cancer [84], it is conceivable that there is an increased chance for suicide among these patients caused not only by the cancer itself, but also based on personal predisposition. Comparing the SMRs of other smoking-related cancers (i.e., oral cavity, larynx, esophagus) to sites without specific associations with tobacco use (i.e., skin, thyroid, brain), smoking-associated cancer patients have an elevated risk for suicide, although some cancer types without a clear association with smoking often share a uniformly fatal prognosis [4]. Most likely, both the strong association with smoking and the particularly poor prognosis, even when compared to other histological lung cancer subtypes [85,86] contribute to the high risk of suicide among patients with SCLC.

Moreover, apart from tumor type and stage, performance status in everyday living is the most important factor when it comes to prognosis, independent from histological subtype, which often happens to be decreased in (ex-)smokers from the onset [87].

Our study has several limitations. Data collection may have varied according to the respective registry. However, suicides are most likely reliably documented. Suicide as a “private issue” may also be culturally charged (i.e., partly regarded as a heroic act in the Japanese culture whereas seen as immoral in areas influenced by Christian religion), which may have influenced registration across different registries. Furthermore, data are unavailable from the continents of Africa and Australia.

In addition, we had to deal with various adjustment factors. However, we were able to ensure at least age-adjusted data as part of our inclusion criteria. We were not able to perform stratified analyses according to smoking status and the presence of depressive symptoms. Furthermore, other well-known risk factors for suicide, such as previous suicide attempts, psychiatric comorbidities, psychiatric treatment or a positive family history for suicide could not be explored as they were not recorded in the primary studies.

The downside of excluding studies because of potential patient data overlap was a restriction of analyzable risk subgroups as shown in Figure 2 and Figure 3. While the dilemma of potential data overlap was handled inconsistently among previous meta-analyses [88,89,90,91], our aim was to assess suicidality in lung cancer patients using the most conservative statistical methodology, in awareness of the consequences mentioned above.

Our study has several important strengths. To date, no other meta-analysis has explored suicidal behaviors specifically in patients with lung cancer. By performing an extensive systematic literature research in five different high-standard databases with broad periods of time, chances of missing further relevant data have been kept small. Our study features objectivity by engaging two different researchers and a third researcher for any disagreements, all acting independently. Additionally, the analyzing process followed a highly standardized procedure. The results of our study are based on a large patient population and numerous subgroup analyses, as well as a sizeable number of different original papers performed in diverse countries.

Furthermore, we followed highly established standards, starting with a structured literature search according to well-founded guidelines and reaching far back in the past, ensuring proper quantitative and qualitative analysis.

## 5. Conclusions

In conclusion, the risk for committing suicide among patients with lung cancer is highly elevated by nearly three- and twofold in comparison to both the general population and to many other cancer entities. The highest risk was observed among patients within the first year after diagnosis. Caregivers should be aware of patients at risk and take individualized clinical decisions when and how to implement specialized psycho-oncologic and psychotherapeutic/psychiatric care. The prompt implementation of a standardized suicidality screening following diagnosis should be considered, particularly if the affected individuals present with any of the aforementioned risk factors.

Further studies should focus on the role of smoking and depressive symptoms on suicidality among lung cancer patients.

## Figures and Tables

**Table 1:** Characteristics of included studies.

N	Author, Year	Time of Recruitment	SMR (95% CI)	Country	Subgroups	*n* Lung Cancer Patients	*n* Suicides	Person- Years
1	Abdel-Rahman et al. 2019 [39]	2000–2010	3.13 (2.83–3.46)	USA	n.a.	348,831	387	n.a.
2	Ahn et al. 2010 [6]	1993–2005	2.89 (2.52–3.30)	Korea	gender, time after diagnosis	72,898	181	n.a.
3	Allebeck et al. 1989 [11]	1962–1979	3.14 (2.47–3.93)	Sweden	gender	n.a.	71	n.a.
4	Allebeck et al. 1991 [30]	1975–1985	3.93 (2.40–6.09)	Sweden	gender	n.a.	17	n.a.
5	Hem et al. 2004 [31]	1960–1997	3.74 (2.77–4.95)	Norway	gender	14,567	46	56,808
6	Henson et al. 2018 [42]	1995–2018	2.57 (2.23–2.97)	England	gender	613,772	184	
7	Innos et al. 2003 [7]	1983–1998	2.17 (1.57–2.92)	Estonia	gender, time after diagnosis, age	n.a.	25	n.a.
8	Kaceniene et al. 2017 [32]	1993–2012	1.94 (1.48–2.55)	Lithuania	gender	n.a.	51	n.a.
9	Kaceniene et al. 2018 [44]	1998–2012	2.17 (1.60–2.95)	Lithuania	gender	19,781	41	79,124
10	Kumar et al. 2017 [40]	2000–2013	3.48 (2.6–4.6)	USA	n.a.	n.a.	137	n.a.
11	Levi et al. 1991 [10]	1976–1987	3.27 (1.20–7.24)	Switzerland	n.a.	n.a.	5	n.a.
12	Lin et al. 2017 [33]	1985–2007	4.00 (3.54–4.51)	Taiwan	gender	n.a.	n.a.	n.a.
13	Louhivuori et al. 1979 [34]	1955–1965	1.29 (0.63–2.36)	Finland	stage	n.a.	9	n.a.
14	Misono et al. 2008 [12]	1973–2002	5.74 (5.30–6.22)	USA	gender	362,163	610	54,131
15	Oberaigner et al. 2014 [8]	1991–2010	4.16 (2.27–6.98)	Austria	gender	5724	14	n.a.
16	Oh et al. 2020 [41]	2000–2016	2.97 (2.69–3.26)	Korea	gender	n.a.	424	n.a.
17	Rahouma et al. 2018 [16]	1973–2013	4.17 (3.87–4.48)	USA	gender, stage, grade, marital status, ethnicity, histology	n.a.	739	n.a.
18	Saad et al. 2018 [9]	2000–2014	5.95 (5.57–6.35)	USA	time after diagnosis, age, stage	798,609	553	n.a.
19	Smailyte et al. 2013 [35]	2001–2009	3.38 (2.22–4.95)	Lithuania	gender	1237	24	9896
20	Tanaka et al. 1999 [43]	1978–1994	2.47 (0.8–5.77)	Japan	n.a.	2976	5	
21	Urban et al. 2013 [36]	1973–2008	4.95 (4.68–5.24)	USA	gender, age, stage, grade, marital status, ethnicity, histology	87,123	1184	n.a.
22	Vyssoki et al. 2015 [37]	1983–2000	3.86 (3.36–4.42)	Austria	n.a.	76,118	208	1,294,014
23	Zaorsky et al. 2019 [38]	1973–2014	11.67 (10.74–12.66)	USA	gender, time after diagnosis	n.a.	583	n.a.
					Total	1,790,027	5498	1,493,973

Abbreviations: n.a.: not available.

## Data Availability

The data that support the findings of this study are available from the corresponding author upon reasonable request.

## Appendix B

**Table A1:** Summary table of meta-regression tests.

Co-Variable		SMR	95% CI	*p*-Value	*q*-Value
Sex				0.03	0.38
	women	3.32	2.92–3.78	0.61	
	men	3.27	2.71–3.95	-	
Stage				0.027	0.38
	early stage cancer	1.36	0.24–7.76	0.67	
	late stage cancer	4.68	1.28–17.14	0.27	
Region				0.03	0.38
	USA	4.17	3.88–4.48	0.15	
	Europe	2.67	2.05–3.47	-	
	Asia	3.31	2.50–4.39	0.38	
Time after diagnosis				<0.001	<0.001
	Within 1 year after diagnosis	5.00	4.11–6.08	<0.001	
	Later than 1 year after diagnosis	1.52	1.21–1.91	-	
Age				0.09	0.91
	>70	3.40	1.70–6.79	0.68	
	60–69	3.23	2.89–3.61	0.89	
	<60	3.09	2.46–3.88	-	

Results are presented for the overall test as well as the comparison of each category compared with the reference category (depicted by a dash ‘–’). *p* value is the summary effect of each subgroup in the meta-analysis; the *q* value is the false discovery rate-adjusted *p* value of the overall test corrected for multiple testing.
